# A two-tiered high-flow nasal cannula approach does not increase intensive care utilization and hospital length of stay in bronchiolitis

**DOI:** 10.1007/s00431-024-05656-7

**Published:** 2024-06-26

**Authors:** Francesca Tirelli, Marco Todeschini Premuda, Giulia Francaviglia, Anna Chiara Frigo, Eugenio Baraldi, Liviana Da Dalt, Silvia Bressan

**Affiliations:** 1 Department of Women’s and Children’s Health, University of Padova, Via Giustiniani 3, 35128 Padua, Italy; 2 Unit of Biostatistics, Epidemiology and Public Health, Department of Cardiac, Vascular, Thoracic Sciences, and Public Health, University of Padova, Via Loredan 18, Padua, Italy

**Keywords:** Bronchiolitis, Two-tiered approach, High-flow nasal canulae oxygen

## Abstract

While concerns about high-flow nasal cannula oxygen (HFNC) overuse and associated increased use of hospital resources are rapidly spreading, a two-tiered approach in its use is recommended by recent bronchiolitis guidelines. However, data on its effects in practice have not been reported. We aimed to analyze the trends in use of HFNC, hospitalizations, length of stay (LOS), and intensive care unit (ICU) admissions for bronchiolitis in a tertiary care center using a two-tiered HFNC approach since its introduction in practice. We retrospectively included data of children < 12 months of age who presented to the Paediatric Emergency Department (PED) and were hospitalized for bronchiolitis at our institution in the epidemic season between October 1st and April 30th during the years 2012–2023 and compared the clinical data across the years. Of the 687 hospitalized children included, 79.9% required oxygen supplementation. Use of HFNC significantly increased since its implementation (from 25% in 2012–2013 to over 60% since 2019–2020, *p* < 0.0001) and was most frequently administered as rescue treatment (in 57.5% of patients). There was no increased trend in ICU admissions (between 1.5% and 10.0% of hospitalizations across seasons, *p* = 0.40), while LOS, after increasing between 2013 and 2016 (medians between 4.0 and 5.4 days), remained stable thereafter (medians between 3.8 and 4.3 days).

*Conclusions*: The use of HFNC according to a two-tiered approach does not appear to be associated with an increase in ICU utilization or LOS.

**Table:** 

**What is Known:** • *Bronchiolitis is one of the most common reasons for hospitalization in infants.* *• Use high-flow nasal canulae oxygen (HFNC) has rapidly spread outside the intensive care unit (ICU) to treat infants with bronchiolitis, although increasing evidence has dampened the initial enthusiasm about their effectiveness.* • *Concerns nowadays are rising about HFNC overuse and associated increased use of hospital resources, including escalation of care to ICU.*
**What is New:** • * A more selective use of HFNC according to a “two-tiered approach”, intended as a second-line rescue treatment in non-severely ill children who fail standard oxygen therapy, is not associated with increased ICU and length of hospital stay.*

**Supplementary Information:**

The online version contains supplementary material available at 10.1007/s00431-024-05656-7.

## Introduction

Bronchiolitis remains one of the most common reasons for hospitalization in infants younger than 1 year of age. Following a dramatic decrease of bronchiolitis cases during the strict public health measures enacted to control the COVID-19 pandemic, an abrupt resurgence of cases was reported after restrictions were lifted [1, 2]. This unexpected surge of bronchiolitis strained hospital bed capacity across continents, often leading to a more intense healthcare burden, including an incremental use of high-flow nasal cannula oxygen (HFNC) [3]. In addition, an increasing HFNC use has been recorded over the years, well before the pandemic. It has been shown that HFNC in moderate-to-severe bronchiolitis can reduce treatment failure more effectively than standard oxygen, but it does not seem to modify the overall disease course. In fact, its early use does not reduce length of stay (LOS), duration of oxygen therapy, and need for intensive care unit (ICU) admission [4, 5]. Moreover, population-based studies have postulated that indiscriminate HFNC use might lead to a raise in ICU admissions [6, 7]. Indeed, it has been hypothesized that an early and too generalized use of HFNC, especially in non-hypoxemic children, might induce a misperception of higher disease severity, inducing to an earlier escalation to intensive care [7]. While concerns about HFNC overuse and associated increased use of hospital resources are rapidly spreading, a two-tiered approach in their use, intended as a second-line rescue treatment in hypoxemic children who fail standard oxygen therapy, is recommended by recent bronchiolitis guidelines [4, 8]. However, data on its effects in practice have not been reported.

We previously showed that a two-tiered approach to HFNC use in our tertiary-care centre for patients with mild-moderate bronchiolitis was associated with low ICU admissions and no adverse outcomes over a limited time window [9]. In the current study, using a single-centre-based hospital registry, we aimed to analyze the trends in HFNC use, LOS, and ICU admissions of children with bronchiolitis across 11 epidemic seasons, including the pre and post COVID-19 pandemic seasons.

## Methods

We extracted data from a hospital registry including children 12 months of age and younger who presented to the Paediatric Emergency Department (PED) and were hospitalized for bronchiolitis at the University Hospital of Padova, between October 1st and April 30th during the years 2012–2023. Children transferred from other centers were excluded as decision on HFNC administration is likely to follow different guidelines in other hospitals. Monthly overall PED visits, as well as visits for bronchiolitis, were also recorded for the study periods. Study data were managed using REDCap (Research Electronic Data Capture).

In our department, HFNC has been used following a two-tiered approach since its introduction in clinical practice in 2011 [10]. Our internal guideline on the management of supplemental oxygen in children with bronchiolitis is reported in the Supplementary Material. Bronchiolitis severity is assessed based on the score reported in Supplementary Material (Table S1).

Data in the registry include demographics, clinical characteristics, data on hospitalization, oxygen supply and modality, treatments received, and need for ICU admission. Data were collected from electronic health records by data abstractors trained by experienced clinician researchers. Missing information occurred only for the variables sex (1 of 687 visits) and oxygen initial modality (1 of 687 visits). Data were analyzed using standard descriptive statistics and the unit of analysis was patient encounter. Comparison of rates across the different seasons was performed using Poisson or Binomial regression, while LOS was compared with Kruskal–Wallis test. Details of these analyses are reported in the Supplementary Material. The tests were two-sided and statistical significance was considered for *p* value < 0.05. SAS 9.4 (SAS Institute Inc., Cary, NC, USA.) for Windows was used for statistical analysis. The study was approved by the Ethical Committee of Padova University Hospital and was conducted in accordance with the Declaration of Helsinki.

## Results

**Fig. 1:**
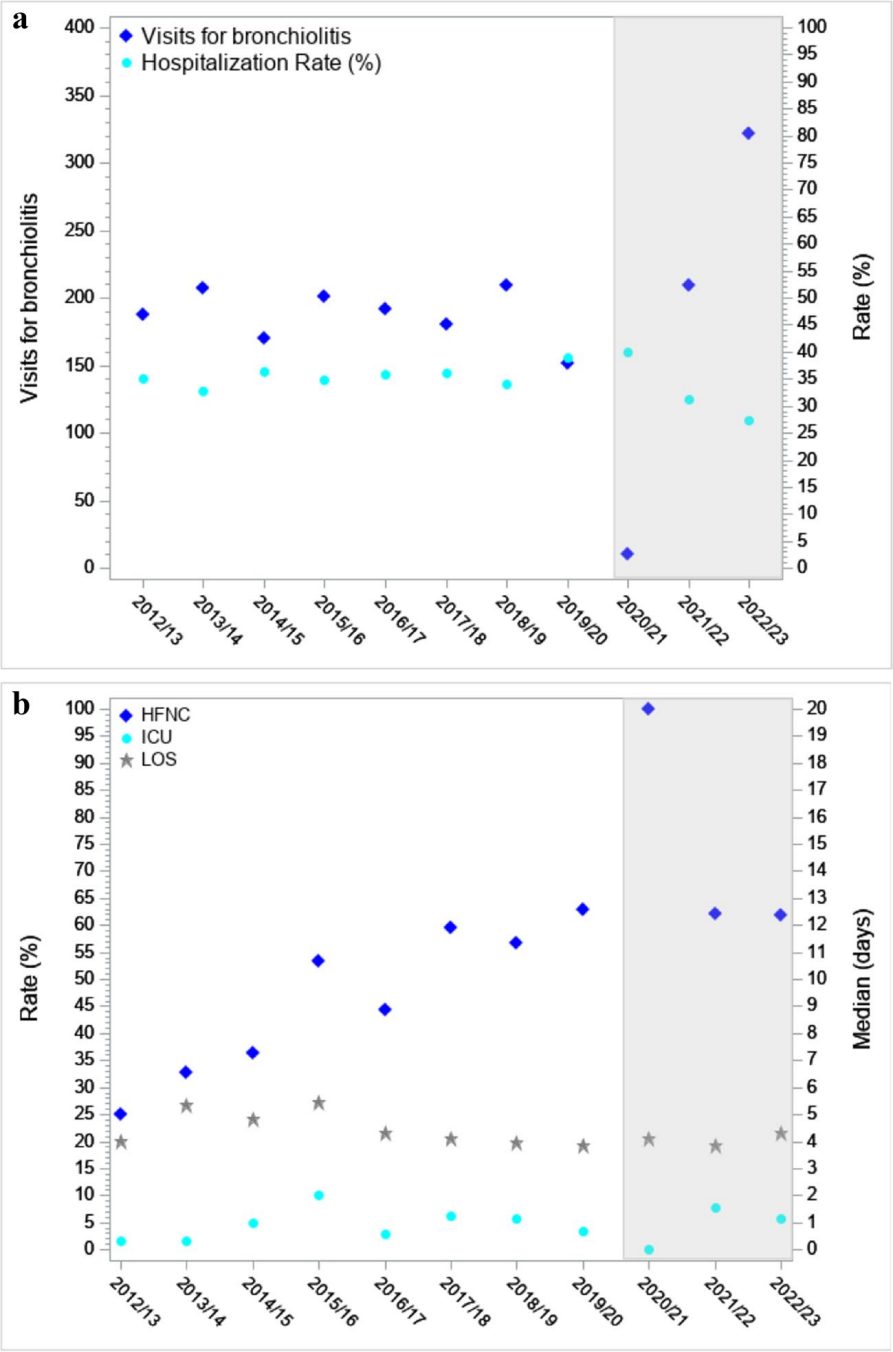
**a** Trends in Pediatric Emergency Department visits and hospitalization for bronchiolitis. The pandemic and post-pandemic seasons are represented within a light grey area. **b** Trends in HFNC use, length of stay, and need for ICU admission. HFNC, high-flow nasal canual; ICU, intensive care unit; LOS, length of hospital stay. LOS is expressed as medians. The pandemic and post-pandemic seasons are represented within a light grey area

During the study period, of 160,788 PED visits, 2040 (1.3%) were for bronchiolitis, of which 797 (39.1%) required hospital admission. Of these, 110 were excluded due to transfer from other centers (Supplementary Fig. 1). The rate of PED visits for bronchiolitis and of hospital admissions over time, and their 95% confidence intervals are reported in Supplementary Tables S3-4 and Fig. 1a, which show the change in epidemiology related to the COVID-19 pandemic and its aftermath. In the post-pandemic seasons, the peak case incidence occurred earlier and concentrated in a shorter period of time compared with the usual peak occurring in December–February. Overall, 46.2% of hospitalizations were recorded in November in the season 2021–2022 and 48.9% in December in the season 2022–2023 (Supplementary Fig. S2).

Of the 687 visits resulting in hospital admission over the study years, 383 (55.8%) were males, the median age was 60 days (IQR 34–117), and the median weight at admission was 4.9 kg (IQR 4.0–6.3). A total of 672 (97.8%) encounters underwent nasopharyngeal testing for viruses. Respiratory syncytial virus (RSV) was isolated in 497 (72.3%) encounters. Rates of RSV-related cases significantly varied over the study seasons between 62.7% (season 2016–2017) and 85.2% (season 2022–2023) (*p* = 0.014). Risk factors for severe bronchiolitis were present in 174 (25.3%) admissions: 125 (18.2%) had prematurity, 32 (4.7%) heart diseases, 18 (2.6%) bronchopulmonary dysplasia, 12 (1.8%) neurological diseases, and 73 (10.8%) other comorbidities. Supplementary Table 2 summarizes the characteristics of patient encounters.

Overall, 548 (79.9%) admissions required oxygen supplementation. Use of HFNC significantly increased since their implementation, from 25.0% of hospitalizations for bronchiolitis in 2012 to approximately 62% since the 2019–2020 season onwards (*p* < 0.0001) (Fig. 1b, Supplementary Table S5). HFNC was most frequently administered as rescue treatment following low-flow oxygen therapy failure (in 57.5% of cases). However, the remaining 42.5% received HFNC as first line oxygen treatment, due to a moderate-severe presentation on initial assessment; this approach also became more common over time, ranging between 55.7% and 61.3% in the seasons 2018–2019 and 2021–2022, to then decrease to 36.4% in the last season.

ICU admission occurred in 34 (5.0%) encounters, including 9 (26.5%) direct admissions from the PED and 25 (73.5%) tranfers to the ICU from the pediatric ward. Admissions to ICU ranged between 1.5% and 10.0% of hospitalizations, with fluctuating values over time, without an increase in trend (*p* = 0.40) (Fig. 1b, Supplementary Table S6). Finally, the median overall LOS was 4.3 days (IQR 3.1–6.1). The median LOS, after a significant increase in the seasons between 2013 and 2016 (medians between 4.8 and 5.4 days), decreased and remained stable during the following seasons (medians between 3.8 and 4.3 days) (*p* = 0.0013) (Fig. 1b, Supplementary Table S7).

## Discussion

This study analyzed the variation in hospital care over time for bronchiolitis in a third-level Pediatric Center over a large observational period of 11 years, following the introduction of HFNC in our practice, and covering the Sars-CoV-2 pandemic and two post-pandemic seasons. Several reports have highlighted an increase in noninvasive ventilation, ICU admissions, and medical costs, in the management of children with bronchiolitis, suggesting this phenomenon to be driven by increasing HFNC utilization [6, 7, 11]. Despite an increase in HFNC use over time in our center, with rates similar to other centers [12], we did not record an increase in ICU admissions or LOS over time. Our internal guideline, limiting HFNC use on the basis of a two-tier approach since HFNC introduction in practice is likely to explain the different results in our center. Although LOS has not increased alongside with increased HFNC utilization, the duration of hospitalization in our study is longer than reported in other centers [12]. A recent study, reporting shorter LOS than ours, has shown that a shorter LOS is associated with a more restrictive use of HFNC, while inpatient weaning protocols do not seem to play a role [12]. While hospital policies and guidelines may limit HFNC use in different ways, it is not clear whether our LOS results may be attributable to a difference in the study population, the healthcare system, or local practice, rather than HFNC use, as its incremental use over the years did not lead to increased LOS. Unfortunately, we could not compare LOS before and after HFNC implementation in clinical practice, as our registry was set up following HFNC introduction and the unavailability of electronic health records before than makes it extremely cumbersome to retrieve data useful for this purpose. Although our more restrictive use of HFNC was not associated with an increased use of ICU resources or of LOS, the actual advantage of HFNC use as recommended by the most recent guidelines remains to be clarified. Future studies should assess the effects of the most recent recommendations, which advise for low-flow oxygen as a first line oxygen supplementation independently of the clinical severity of bronchiolitis on initial assessment [8].

Our study, in line with previous literature [1, 2], also confirms a relevant epidemiological change of bronchiolitis following the Sars-CoV2 pandemic, highlighting a drastic reduction in PED visits and hospitalizations during the pandemic season (2020–2021). Following the pandemic, we observed an anticipated and more intense peak case incidence, concentrating in the months of November and December. Strikingly, during the last 2022–2023 season, the total number of overall bronchiolitis visits and hospitalizations was the highest recorded across the whole observation period. In light of the elevated numbers, we consider that the relatively lower hospitalization rate in the 2022–2023 season was due to saturated hospital bed capacity. As highlighted by the very high rate of HFNC use, only the most severe patients were hospitalized, while different strategies such as prolonged PED observation unit stay or frequent PED revisits for close follow-up were implemented for patients with less severe presentations, but at risk of deterioration.

Our study has some limitations. First, this was a single-center study performed in a tertiary care hospital which serves as hub for a large region in Italy, thus leading to possible overestimation of total bronchiolitis cases, especially in the post-pandemic season. Second, the numbers of overall PED visits were retrieved as aggregate data, which also includes children transferred from other centers; in addition, we could not differentiate between first self-presentations and scheduled PED revisits, which occurred more frequently in the last season due to a change in management dictated from a saturated hospital bed capacity. Third, as we used a hospital-based registry set-up in 2012, covering the traditional epidemic season (from October to April), we were not able to capture the patients with bronchiolitis who presented outside the defined data collection period. Given the change in epidemiology following the first pandemic season, we were not able to include patients presenting earlier than the usual epidemic season. However, given that nearly 50% of patients presented in the months of November for the season 2021–2022 and December for the season 2022–2023 (as reported in the “Results” section), we believe our results are unlikely to be significantly affected by the patients we missed.

In conclusion, our study shows that a more restrictive use of HFNC in pediatric wards, based on a two-tiered approach, does not appear to be associated with an increase use of ICU care and did not negatively impact LOS. As the bronchiolitis epidemiological landscape remained altered 2 years after the Sars-CoV-2 pandemic with important implications with respect to hospital contingency plans and organization of alternative management pathways, continuous epidemiological surveillance of bronchiolitis is necessary to optimize hospital preparedness and management protocols, to strengthen prevention strategies and monitor appropriate HFNC use, awaiting for universal prophylaxis to change the current landscape.

### Supplementary Information

Below is the link to the electronic supplementary material.

Supplementary file1 (DOCX 135 KB)

## Data Availability

The data that support the findings of this study are not openly available due to reasons of sensitivity and are available from the corresponding author upon reasonable request.
