# Supplementary material for: A two-tiered high-flow nasal cannula approach does not increase intensive care utilization and hospital length of stay in bronchiolitis
Source: Eur J Pediatr. 2024 Jun 26;183(9):4133–7. doi: 10.1007/s00431-024-05656-7 (PMC11322270; doi:10.1007/s00431-024-05656-7)
Supplement: Supplementary file 1 — Supplementary file1 (DOCX 135 KB) [file 431_2024_5656_MOESM1_ESM.docx]

**SUPPLEMENTARY MATERIAL**

**SUPPLEMENTARY METHODS**

**Oxygen administration protocol for infants with bronchiolitis used at our Center:** Children with oxygen saturation <92% on room air receive low-flow oxygen, delivered at less or equal to 2 L/min if they have no signs of moderate-severe respiratory distress. HFNC is administered as rescue therapy for infants who deteriorate, based on clinical assessment, while on low-flow oxygen ^1^ or as first line treatment for those infants who have moderate-severe respiratory distress based on the score we use (see Table S1). HNFC administration is provided if infants present a moderate-severe respiratory distress based on the score we use, at any time during the assessment in the ED or during hospitalization. The maximum HFNC flow used in our ward is 2 L/kg/min, with FiO2 adjusted to achieve an oxygen saturation equal to or above 92- 94%. In the presence of severe respiratory distress and/or desaturation despite maximization of HFNC parameters, transfer to ICU is discussed with the ICU team. Children are discharged from hospital when oxygen saturation remains steadily above 92% without oxygen supplementation.

^1^Daverio M, Da Dalt L, Panozzo M, Frigo AC, Bressan S. A two-tiered high-flow nasal cannula approach to bronchiolitis was associated with low admission rate to intensive care and no adverse outcomes. Acta Paediatr. 2019;108 :2056-2062.

**Poisson and binomial regression models:** The effect of the different seasons (independent variable) on bronchiolitis visits rate and ICU admission rate (dependent variable) was modeled through Poisson regression model. While for bronchiolitis related hospitalizations rate, RSV rate, and HFNC use (dependent variable) the effect of the seasons (independent variable) was modeled with binomial regression. The rates in each season were estimated with 95% confidence intervals derived from the model applied. Length of hospitalization (LOS) (dependent variable) was analyzed with non-parametric Kruskall-Wallis test for testing the season effect (dependent variable). The median LOS was estimated for each season with 95% confidence intervals using the distribution-free method.

**SUPPLEMENTARY TABLE S1**

Bronchiolitis severity criteria

|  | **Mild** | **Moderate** | **Severe** |
| --- | --- | --- | --- |
| **Respiratory rate** | <60 breaths per minute | 60 – 70 breaths per minute | >70 breaths per minute |
| **Dyspnea** | Mild chest retractions | Moderate chest retractions/ Suprasternal retractions / Nasal flaring | Moderate chest retractions/ Suprasternal retractions / Nasal flaring /Grunting / Head Bobbing |
| **O2 saturation** | >94% in room air | 90 – 94% in room air | <90% in room air |
| **Feeding** | Normal / Mild reduction | 50 – 75% than normal intake | <50% than normal intake / absent |
| **Apnea** | No | Brief episodes | Major episodes |

When criteria from different levels of severity are present, the greater severity is considered.

Reduction in feeding is considered based on usual intake of milk or solid food as reported by caregivers.

Minor episodes of apnea are defined as cessation of breathing <20 seconds, without bradycardia/cyanosis. Major episodes of apnea are defined as documented cessation of breathing >20 seconds and/or accompanied by bradycardia/cyanosis.

**SUPPLEMENTARY TABLE S2**

Demographic and clinical characteristics of included patient encounters.

|  | **n=687** |
| --- | --- |
| Age in days (median, range) | 60 (3 – 365) |
| Age ≤90 days (*n,%*) | 445 (64.8%) |
| Sex (male) (*n,%*) | 383 (55.8%) |
| Weight in Kg (median, range) | 4.9 (4.0 – 6.3) |
| History of prematurity (*n,%*) | 125 (18.2%) |
| Comorbidities (*n,%*)  Heart diseases  Bronchopulmonary dysplasia  Neurological diseases  Other | 32 (4.7%)  18 (2.6%)  12 (1.8%)  73 (10.8%) |
| RSV positive (*n,%*) | 497 (72.3%) |

**SUPPLEMENTARY TABLE S3**

**Results of Poisson regression model for rate of bronchiolitis related visits**

| **Season** | **Rate of bronchiolitis related visits (%)** | **95% Confidence Interval** | |
| --- | --- | --- | --- |
| **2012/13** | 1.332 | 1.155 | 0.01537 |
| **2013/14** | 1.483 | 1.294 | 1.699 |
| **2014/15** | 1.113 | 0.9576 | 1.293 |
| **2015/16** | 1.312 | 1.143 | 1.507 |
| **2016/17** | 1.279 | 1.110 | 1.473 |
| **2017/18** | 1.169 | 1.010 | 1.353 |
| **2018/19** | 1.313 | 1.147 | 1.504 |
| **2019/20** | 1.207 | 1.029 | 1.415 |
| **2020/21** | 0.1103 | 0.0593 | 0.2050 |
| **2021/22** | 1.321 | 1.153 | 1.513 |
| **2022/23** | 1.758 | 1.576 | 1.961 |

**Season effect p<0.0001 (Poisson model)**

**SUPPLEMENTARY TABLE S4**

**Results of binomial regression model for bronchiolitis related hospitalization rate**

| **Season** | **Bronchiolitis related Hospitalization rate (%)** | **95% Confidence Interval** | |
| --- | --- | --- | --- |
| **2012/13** | 35.11 | 28.62 | 42.19 |
| **2013/14** | 32.85 | 26.80 | 39.53 |
| **2014/15** | 36.47 | 29.58 | 43.96 |
| **2015/16** | 34.83 | 28.56 | 41.67 |
| **2016/17** | 35.94 | 29.47 | 42.96 |
| **2017/18** | 36.11 | 29.43 | 43.38 |
| **2018/19** | 33.97 | 27.87 | 40.65 |
| **2019/20** | 38.82 | 31.40 | 46.78 |
| **2020/21** | 40.00 | 15.83 | 70.26 |
| **2021/22** | 31.10 | 25.19 | 37.69 |
| **2022/23** | 27.33 | 22.74 | 32.46 |

**Season effect p=0.4035 (Binomial model)**

**SUPPLEMENTARY TABLE S5**

**Results of binomial regression model for rate of HFNC use**

| **Season** | **HFNC Rate (%)** | **95% Confidence Interval** | |
| --- | --- | --- | --- |
| **2012/13** | 25.00 | 15.40 | 37.90 |
| **2013/14** | 32.76 | 21.97 | 45.74 |
| **2014/15** | 36.36 | 24.81 | 49.74 |
| **2015/16** | 53.33 | 40.77 | 65.49 |
| **2016/17** | 44.44 | 31.87 | 57.78 |
| **2017/18** | 59.57 | 45.15 | 72.52 |
| **2018/19** | 56.60 | 43.11 | 69.19 |
| **2019/20** | 62.79 | 47.62 | 75.80 |
| **2020/21** | 100.00 | NOT ESTIMABLE | NOT ESTIMABLE |
| **2021/22** | 62.00 | 47.96 | 74.28 |
| **2022/23** | 61.76 | 49.76 | 72.49 |

**Season effect p<0.0001 (Binomial model)**

**SUPPLEMENTARY TABLE S6**

**Results of Poisson regression model for ICU admission rate**

| **Season** | **ICU Admission Rate (%)** | **95% Confidence Interval** | |
| --- | --- | --- | --- |
| **2012/13** | 1.515 | 0.2134 | 10.76 |
| **2013/14** | 1.471 | 0.2072 | 10.44 |
| **2014/15** | 4.839 | 1.561 | 15.00 |
| **2015/16** | 10.00 | 4.767 | 20.98 |
| **2016/17** | 2.899 | 0.7249 | 11.59 |
| **2017/18** | 6.154 | 2.310 | 16.40 |
| **2018/19** | 5.634 | 2.114 | 15.01 |
| **2019/20** | 3.390 | 0.8478 | 13.55 |
| **2020/21** | 0.00 | NOT ESTIMABLE | NOT ESTIMABLE |
| **2021/22** | 7.692 | 3.202 | 18.48 |
| **2022/23** | 5.682 | 2.365 | 13.65 |

**Season effect p= 0.3979 (Poisson model)**

**SUPPLEMENTARY TABLE S7**

**Results of binomial regression model for rate of length of hospital stay**

| **Season** | **Median LOS (days)** | **95% Confidence Interval** | |
| --- | --- | --- | --- |
| **2012/13** | 3.95833 | 3.66667 | 4.54167 |
| **2013/14** | 5.25000 | 4.16667 | 6.12500 |
| **2014/15** | 4.79167 | 3.87500 | 5.79167 |
| **2015/16** | 5.37500 | 4.70833 | 6.62500 |
| **2016/17** | 4.31250 | 3.70833 | 5.45833 |
| **2017/18** | 4.10417 | 3.70833 | 4.70833 |
| **2018/19** | 3.89583 | 3.66667 | 4.75000 |
| **2019/20** | 3.75000 | 3.04167 | 4.50000 |
| **2020/21** | 4.12500 | 3.12500 | 5.00000 |
| **2021/22** | 3.77083 | 3.12500 | 4.70833 |
| **2022/23** | 4.25000 | 3.91667 | 4.91667 |

**Season effect p= 0.0013 (Kruskal-Wallis test)**

**SUPPLEMENTARY FIGURE 1**

Flowchart of patient enrollment

**SUPPLEMENTARY FIGURE S2**

Bronchiolitis hospitalizations per month over the study seasons
